# In Situ Measurement of NO, NO_2_, and H_2_O in Combustion Gases Based on Near/Mid-Infrared Laser Absorption Spectroscopy

**DOI:** 10.3390/s22155729

**Published:** 2022-07-31

**Authors:** Jing Li, Renjie Li, Yan Liu, Fei Li, Xin Lin, Xilong Yu, Weiwei Shao, Xiang Xu

**Affiliations:** 1 Key Laboratory of Advanced Energy and Power, Institute of Engineering Thermophysics, Chinese Academy of Sciences, Beijing 100190, China; lijing2020@iet.cn (J.L.); shaoww@iet.cn (W.S.); xuxiang@mail.etp.ac.cn (X.X.); 2 School of Engineering Science, University of Chinese Academy of Sciences, Beijing 100049, China; lirenjie@imech.ac.cn (R.L.); xlyu@imech.ac.cn (X.Y.); 3 State Key Laboratory of High Temperature Gas Dynamics, Institute of Mechanics, Chinese Academy of Sciences, Beijing 100190, China; lifei@imech.ac.cn (F.L.); linxin_bit@imech.ac.cn (X.L.)

**Keywords:** NO_x_ emission, multispecies sensor, in situ measurements, wavelength modulation spectroscopy

## Abstract

In this study, a strategy was developed for in situ, non-intrusive, and quantitative measurement of the oxides of nitrogen (NO and NO_2_) to describe emission characteristics in gas turbines. The linear calibration-free wavelength modulation spectroscopy (LCF-WMS) approach combined with the temperature profile-fitting strategy was utilized for trace NO and NO_2_ concentration detection with broad spectral interference from gaseous water (H_2_O). Transition lines near 1308 nm, 5238 nm, and 6250 nm were selected to investigate the H_2_O, NO, and NO_2_ generated from combustion. Experiments were performed under different equivalence ratios in a combustion exhaust tube, which was heated at 450–700 K, with an effective optical length of 1.57 m. Ultra-low NO_x_ emissions were captured by optical measurements under different equivalence ratios. The mole fractions of H_2_O were in agreement with the theoretical values calculated using Chemkin. Herein, the uncertainty of the TDLAS measurements and the limitation of improving the relative precision are discussed in detail. The proposed strategy proved to be a promising combustion diagnostic technique for the quantitative measurement of low-absorbance trace NO and NO_2_ with strong H_2_O interference in real combustion gases.

## 1. Introduction

Combined-cycle gas turbines have generated more than 22% of the world’s electricity [1]. The percentage in the USA is 43%. With the progress of materials and cooling technology, the temperature of combustion chambers is gradually increasing, improving the combustion efficiency while promoting the emission of nitrogen oxides (NO_x_) in parallel [2]. NO_x_ molecules, mainly nitrogen monoxide (NO) and nitrogen dioxide (NO_2_), have a wide range of health and environmental impacts [3,4,5,6]. Given the detrimental effects of NO_x_ and the demand for higher efficiency, how to address the conflict between increasing the combustion temperature and reducing NO_x_ emission has been an important issue in gas turbine technology research. A series of increasingly stringent regulatory standards have been released to control the NO_x_ emission of combustion-based devices [7,8]. As a result, a reliable in situ and high temporal resolution measurement technique is required to monitor ultra-low NO_x_ emissions, evaluate the combustion organization method, and analyze the influencing factors of emissions [9,10].

Numerous techniques have been developed to quantify the species and concentration of combustion products in recent years, such as chemiluminescence detectors (CLDs), electrochemical gas sensors, chemiresistive gas sensors, Fourier transform infrared spectroscopy (FTIR), non-dispersive infrared (NDIR), and tunable diode laser absorption spectroscopy (TDLAS). These techniques can be broadly categorized into the chemical method and the optical method, varying significantly in their accuracy and interference immunity. For the chemical method, electrochemical gas sensors and chemiresistive gas sensors are well-established techniques for monitoring trace-level concentrations of industrial gases [11,12]. However, the gas sensing properties are strongly influenced by the surrounding environment and the nature of the interacting gases, especially the presence of high relative humidity in combustion environments [13,14]. CLD analyzers have been recommended by SAE International to guide the analysis of NO_x_ emission due to their high accuracy and simplicity [15], but they require pre-processing for cooling and decompression due to their inability to endure high temperatures and pressures. Optical methods use specific spectral information to identify gases and avoid disturbing the flow field. FTIR spectrometers have the advantage of distinguishing multiple gases, but their low light source power demands an increase in integration time to improve the signal-to-noise ratio (SNR) [16,17,18]. NDIR is well known for its good sensitivity, but it suffers from interfering species [19]. For gas turbines, a high temperature and pressure, as well as various interferences, are present in the combustion exhaust. These issues pose significant challenges for the quantitative and accurate measurement of trace NO_x_ in harsh environments.

TDLAS has good sensitivity and quantitative performance and is one of the most promising approaches at present [20,21]. It has been successfully applied to trace gas detection in ambient environments [22,23,24]. However, only a few studies have been reported on trace gas detection in harsh environments. Chao et al. [25] recorded the concentration of NO in combustion exhaust using transitions near 1912 cm^−1^ and 1926 cm^−1^ at 600 K based on direct absorption spectroscopy (DAS). Almodovar et al. [26] presented temperature and NO concentration sensing in high-temperature gases using a pair of quantum cascade lasers (QCLs) near 5 µm. Diemel et al. [27] developed a direct absorption NO sensor (transition at 1929.03 cm^−1^) for combustion exhaust gas. The detection limit was 30 ppm with a 10 ms response time at 800 K. In the mentioned applications, the effect of interfering species can be neglected by selecting suitable absorption lines based on the DAS method, due to the strong absorbance of NO. Sur et al. [28] demonstrated NO_2_ detection of 1.45 and 1.6 ppm at elevated temperatures in combustion exhaust. The first-harmonic-normalized, second-harmonic detection wavelength modulation spectroscopy (2*f*/1*f*-WMS) method [29] was used to probe the selected transitions near 1599.9 cm^−1^. The seeding NO_2_ was detected based on the background signal collected from combustion gases, which not only reduced the interference from gaseous water (H_2_O) but also ignored the NO_2_ generated from the combustion process.

These works confirmed the potential of mid-infrared TDLAS technology in combustion emission diagnosis. However, few studies have focused their attempts on the mid-infrared-based detection of trace NO and NO_2_ generated from combustion and considered interference from other absorption components, such as water and methane (CH_4_). The influence of the neighboring H_2_O spectral features becomes significant as the temperature increases, making it difficult to obtain reliable non-absorbing baselines. Despite the fact that the dependence on the baseline can be eliminated by using the 2*f*/1*f*-WMS approach [30,31], challenges still remain. In the application of combustion diagnostics, a uniform distribution of temperature is commonly assumed along the line of sight (LOS) [32]. This results in the harmonic signals being further distorted due to the nonlinear coupling of the characteristics of the non-uniform flow field and laser parameters, which increases the difficulty of resolving harmonic signals. Recently, linear calibration-free wavelength modulation spectroscopy (LCF-WMS) has been reported [33,34], which is suitable for trace gas detection in non-uniform fields. However, these works were primarily concerned with the performance of a single component, which cannot capture overall NO_x_ emissions. The main objective of this paper was to achieve in situ and quantitative diagnosis of ultra-low NO_x_ emissions under strong absorption interference from H_2_O in combustion exhaust.

In this work, a new strategy is presented to realize the detection of trace NO and NO_2_ with a low signal-to-noise ratio (SNR) despite the strong H_2_O interference. The strategy is based on the LCF-WMS method, combined with temperature profile-fitting, to eliminate the effects of water interference by resolving overlapped harmonic signals. A near-infrared distributed feedback (DFB) laser near 1.3 µm for H_2_O and two mid-infrared quantum cascade lasers (QCLs) near 5.2 µm for NO and 6.25 µm for NO_2_ were employed to probe the selected absorption lines. The validation experiments were performed under different equivalence ratio conditions in a combustion exhaust tube. The proposed method proves not only the capability of trace gas detection in combustion gases under low absorbance and high excess noise levels, but also the capability of quantitative measurements in a non-uniform flow field. The novelty of the current detection strategy lies in the following:

1. The fourth harmonic of the linear calibration-free wavelength modulation spectroscopy (LCF-WMS-4*f*) method for in situ NO_2_ measurement in combustion exhaust at a high temperature;
2. The demonstration of ppm-level NO and NO_2_ detection with high H_2_O interference in a non-uniform flow field.

## 2. TDLAS Diagnostics for NO and NO_2_

The theory of laser absorption spectroscopy is well understood and has been published previously [33,34,35,36]. The principles and techniques of the LCF-WMS method are briefly reviewed in Section 2.1 to define terms and guide the discussion. The selection of transition lines for three components is introduced in Section 2.2. The strategy of NO and NO_2_ detection under high H_2_O interference is shown in Section 2.3.

### 2.1. Fundamentals of Linear Calibration-Free Wavelength Modulation Spectroscopy

When the collimating laser beam passes through a uniform gas medium, the spectral transmissivity $\tau_{\nu }$ is defined according to the ratio between the transmitted beam intensity $I_{t}$ and the incident beam intensity $I_{0}$, which is described by the Beer–Lambert law:

(1) $$\tau_{\nu }={\left(\frac{I_{t}}{I_{0}}\right)}_{\nu }=\exp\left(-\alpha_{\nu }\right)=\exp\left(-k_{\nu }L\right)$$

where $\alpha_{\nu }$ is the spectral absorbance at frequency *ν*, *L* (cm) is the optical path length, and $k_{\nu }$ (cm^−1^) is the spectral absorption coefficient, which is given by

(2) $$k_{\nu }=PXS\left(T\right)\phi_{\nu }$$

where *P* (atm) is the gas pressure, *X* is the mole fraction of the absorbing gas species, *S*(*T*) (cm^−2^ atm^−1^) is the temperature-dependent line strength, and $\phi_{\nu }$ is the line shape function. The line shape function $\phi_{\nu }$ (cm) can be normalized, and therefore the integral of $\phi_{\nu }$ over the entire frequency range is equal to one.

The wavelength of the laser is tuned using a combination of a high-frequency sinusoidal modulation and a lower-frequency scan signal. The frequency modulation and intensity modulation can be expressed as

(3) $$\nu \left(t\right)=\bar{\nu }\left(t\right)+a\left(t\right)\cos\left(2\pi ft+\psi \right)$$

(4) $$I_{0}\left(t\right)={\bar{I}}_{0}\left(t\right)\left[1+\overset{\infty }{\underset{m=1}{\sum }}i_{m}\left(t\right)\cos\left(m\times 2\pi ft+\psi_{m}\right)\right]$$

where $\bar{\nu }\left(t\right)$ (cm^−1^) is the center frequency of the slower wavelength scan signal while the laser is modulated at frequency f, a(t) (cm^−1^) is the modulation depth, and ψ is the frequency modulation phase; ${\bar{I}}_{0}\left(t\right)$ is the incident laser intensity without modulation, $i_{m}\left(t\right)$ is the *m*-th Fourier coefficient of the laser intensity, and $\psi_{m}$ is the phase of the *m*-th laser intensity modulation.

The logarithm of the transmitted laser intensity can be written as

(5) $$\begin{array}{c} \ln\left(I_{t}\left(t\right)\right)=\ln\left(G{\bar{I}}_{0}\left(t\right)\right)+\ln\left[1+\overset{\infty }{\underset{m=1}{\sum }}i_{m}\left(t\right)\cos\left(m\times 2\pi ft+\psi_{m}\right)\right]\\ -PXS\left(T\right)L\times \phi_{\nu }\left[\bar{\nu }\left(t\right)+a\left(t\right)\cos\left(2\pi ft+\psi \right),T,P,X\right] \end{array}$$

After the logarithmic calculation, the transmitted light intensity consists of three parts: the optical-electronic gain intensity term, the intensity modulation term, and the absorption signal term. The harmonic signal is extracted from $\ln(I_{t}\left(t)\right)$ by lock-in amplifier, and the *k*-th absorption harmonic signal becomes

(6) $$S_{k}^{a}=\frac{1}{2}PXS\left(T\right)L\times \left|\overset{\infty }{\underset{g=0}{\sum }}\left({\frac{{\left(a\left(t\right)/2\right)}^{k+2g}}{g!\left(k+g\right)!}\frac{d^{k+2g}\phi_{v}}{d\nu^{k+2g}}|}_{\nu =\bar{\nu }}\right)\right|$$

After subtracting the background, harmonic signals are theoretically independent of the laser intensity characteristics. The measured harmonics are only related to the time–frequency relationship (the line shape derivative $\frac{d^{k+2g}\phi_{v}}{{d\nu }^{k+2g}}$ and the modulation depth a(t)) and the integrated absorbance. During the experiments, the time–frequency relationship can be obtained using a Fabry–Perot interferometer. The signals detected are only associated with the spectral features integrated along the LOS.

Owing to the decoupling of the laser characteristics and the integrated absorbance, the influence of gas properties can be clearly reflected in the harmonics. This approach allows the numerical simulation of large absorption harmonics without considering the effect of laser intensity modulation characteristics. Therefore, the LCF-WMS approach is suitable for trace gas detection in spite of the strong, broad spectral H_2_O interference in non-uniform fields for the following reasons: compared with the DAS approach, it eliminates the dependence on the baseline and suppresses the low-frequency noises; compared with the normalized WMS approach, it decouples the characteristics of the light source and the flow field, making the measured signals related to the spectral features integrated along the line of sight (LOS).

### 2.2. Wavelength Selection

The selection of absorption transitions is critical from the viewpoint of multi-wavelength sensor design. Considering the composition of typical combustion products, H_2_O takes a large proportion (~10%), while NO_x_ molecules are trace components (~ppm). For the detection of NO and NO_2_ generated from combustion, spectroscopic signals can be affected by the blended neighboring features and interfering species spectrum, which decreases the SNR and influences the measurement accuracy. These require transition lines with stronger absorption and weaker interference from the other combustion species [37,38,39]. To ensure a sufficient SNR of the absorption signal, the peak absorbance should be above 0.001.

**Figure 1:**
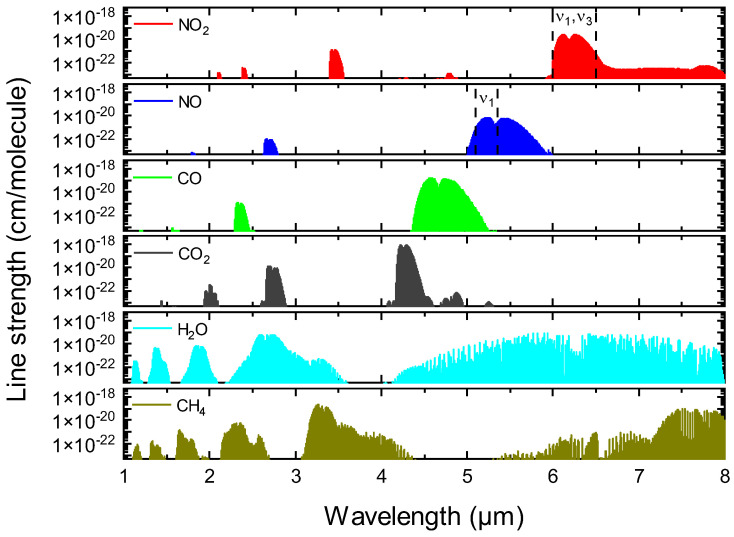
Absorption line strength for near- and mid-infrared bands of typical combustion exhaust at 600 K based on HITRAN 2020 [40]. Transitions with a line strength of less than 5 × 10^−24^ cm/molecule are not shown.

Figure 1 shows a broadband spectral simulation of typical components in near- and mid-infrared bands at a representative exhaust temperature of 600 K based on the HITRAN database [40]. The fundamental vibration band of NO near 5.2 µm and NO_2_ near 6.25 µm holds the most promising candidates, which has the strongest absorption band and only primary interference from H_2_O. In this circumstance, it is important to acquire an accurate H_2_O concentration for the following spectral resolution. The overtone and combination bands within 1.3–1.5 µm were investigated for H_2_O sensing because of the maturity of near-infrared DFB lasers.

**Figure 2:**
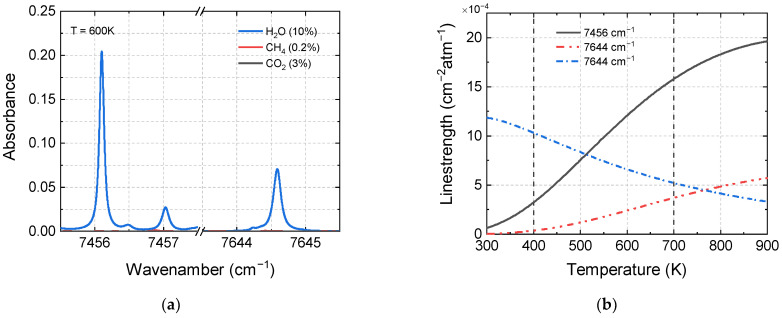
Absorbance simulation of three candidate H_2_O transitions (**a**) and the corresponding line strength varying from 300 to 900 K (**b**). *P* = 1 atm, *L* = 2 m, T = 600 K, *X*_H2O_ = 10%, *X*_CH4_ = 0.2%, *X*_CO2_ = 3%.

According to the aforementioned criteria, the absorption lines in the fundamental band for NO and NO_2_, as well as the overtone and combination band of H_2_O, were investigated. For H_2_O, several candidates (7456.1 cm^−1^, 7457 cm^−1^, and 7644.6 cm^−1^) are plotted in Figure 2a. The absorption line at 7644.6 cm^−1^ was finally selected for the following reasons: (1) 7644.6 cm^−1^ is well isolated; (2) this transition has negligible interference from other absorption species; (3) compared with the strongest absorption line, 7456.1 cm^−1^, this transition has a smaller variation in the line strength within the temperature range of 400–700 K (shown in Figure 2b), which is less sensitive to temperature changes and more suitable for non-uniform field measurements. The validation of the line strength of the selected H_2_O transitions was carried out in an ambient environment. The deviation between the measured line strength and the HITRAN database was less than 8%, while the uncertainty of the spectral parameter was in the range of 5–10%.

**Figure 3:**
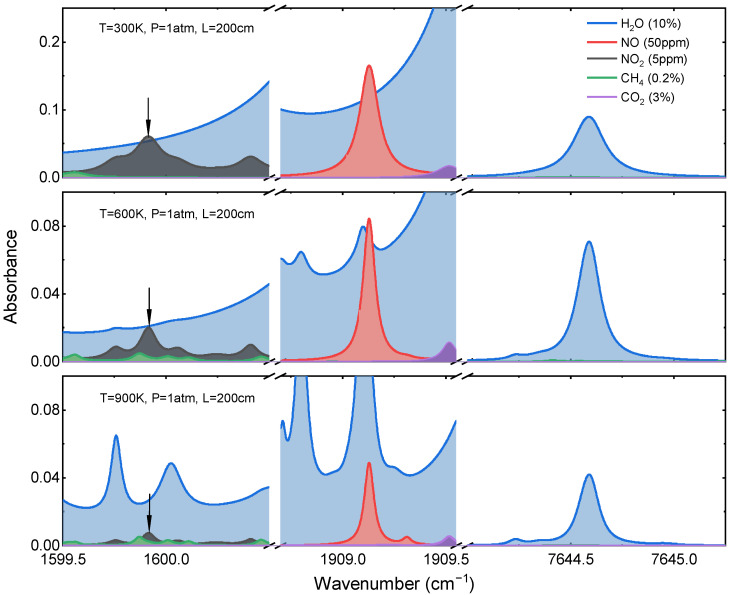
Simulated absorption spectra of the selected H_2_O, NO, and NO_2_ transitions near 1.31, 5.2, and 6.25 µm for typical combustion exhaust: *X*_H2O_ = 10%; *X*_NO_ = 50 ppm; *X*_NO2_ = 10 ppm; *X*_CH4_ = 0.2%; *X*_CO2_ = 3%; *P* = 1 atm; *L* = 2 m; *T* = 300, 600, and 900 K.

The promising transitions near 1909.13 cm^−1^ and 1599.9 cm^−1^ were selected for NO and NO_2_, respectively. The line strength of the selected NO lines was validated in [41], and it agrees with the uncertainty provided by the HITEMP database (5–10% uncertainty) [42]. For NO_2_ candidates near 1599.9 cm^−1^, a reliable collisional broadening parameter database was established by Sur et al. [28]. The simulated spectra of typical components near the target lines are plotted in Figure 3, which indicates that the H_2_O absorptions have major interference from those of NO and NO_2_. In both spectrum regions, the absorption features of H_2_O are smooth and weak at the intermediate temperature. However, several new water spectral features appear with the temperature elevation, making the problem more complicated. In such circumstances, the interference from weak, high-internal-energy water vapor transitions cannot be eliminated by wavelength selection. The strategy for the spectral resolution is discussed in the next section. It must be mentioned that CH_4_ has negative influences on the detection of NO_2_. The influence of CH_4_ becomes significant along with the temperature increases.

### 2.3. NO and NO_2_ Detection under High H_2_O Interference

Broad spectral interference presents great challenges for trace NO and NO_2_ detection. Additional research needs to be conducted to minimize the effect of H_2_O interference on NO and NO_2_ measurements. First of all, multispecies detections are required to obtain the mole fraction of the mixture. Then, the influence of each component on the spectral feature twisting needs to be quantified. Lastly, the technique should have high detection sensitivity to catch the low absorbance of trace gases.

H_2_O concentrations in combustion gases are obtained based on near-infrared TDLAS sensing. With a known concentration, H_2_O absorbance near the selected NO_x_ absorption lines can be calculated by using spectral parameters taken from the HITRAN database [40]. Based on the LCF-WMS approach, the overlapped harmonic signals are resolved by taking into account both the spectral features of the target (NO_x_) and interfering species (H_2_O). Considering the non-uniformity of the flow field, the strategy of temperature profile fitting was adopted in this study.

The second harmonic was recommended due to its higher SNR compared to other order harmonics in the literature [33]. Accordingly, the second harmonic was used for NO and H_2_O detection in the present study. However, it is more difficult to model and resolve the NO_2_ spectrum under the presence of blended neighboring features and the broad spectral interference from H_2_O, especially when H_2_O absorbance is of a magnitude equivalent to NO_2_. Previous works have demonstrated the potential of higher harmonics in the application of broad absorbance [43,44]. In this work, we used the fourth-harmonic signal for NO_2_ detection due to its H_2_O sensitivity reduction.

**Figure 4:**
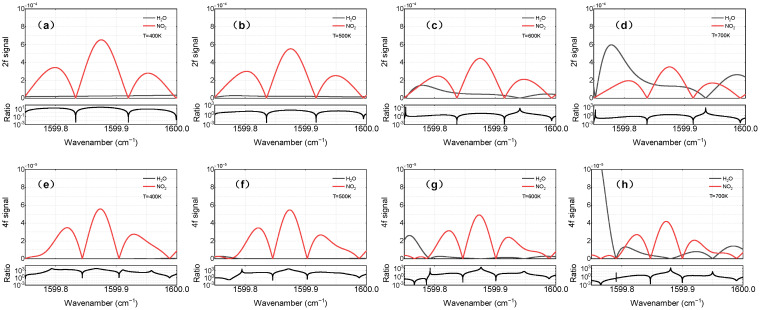
Simulations of 1.5 ppm NO_2_ (red) and 15% H_2_O (black) in the typical exhaust temperature range of 400–700 K, *P* = 1 atm, L = 157.5 cm. The upper line (**a–d**) shows the 2*f* line shapes; the lower line (**e–h**) shows the 4*f* line shapes.

**Figure 5:**
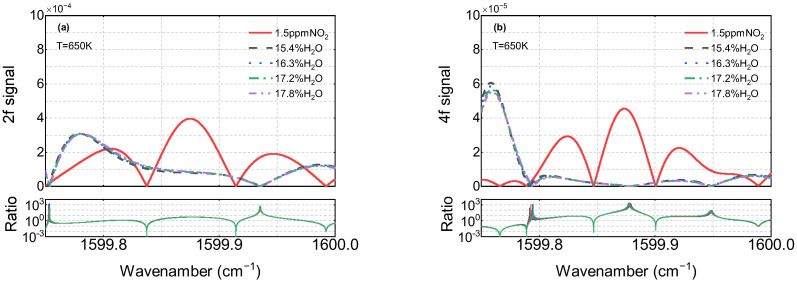
Simulation of 1.5 ppm NO_2_ in 15.4–17.8% H_2_O and the corresponding second harmonic (**a**) and fourth harmonic (**b**). *P* = 1 atm, L = 157.5 cm, T = 650 K.

It needs to be mentioned that the simulated harmonics can directly reflect gas properties, given the decoupling of the laser intensity and flow field characteristics. Figure 4 shows the numerical simulation of the second and fourth harmonics in typical combustion exhaust conditions (NO_2_~1.5 ppm, H_2_O~16%) in the temperature range of 400–700 K. Apparently, the line shape of H_2_O is very sensitive to temperature fluctuations. To evaluate the significance of water vapor interference, the ratio of the NO_2_ to H_2_O harmonic signals is used. When the ratio is higher, it indicates that the influence of H_2_O is greater. In contrast with the second harmonic, the central lobe of the fourth harmonic (between 1599.85 cm^−1^ and 1599.92 cm^−1^) is almost unaffected by water vapor. According to the numerical results shown in Figure 5, within the typical mole fraction range of 15.4–17.8% in combustion gases, the H_2_O concentration has a limited effect on the line shape, and the harmonic performance of the central lobe is in accordance with the above-mentioned phenomenon.

In the previous study on mid-infrared NO_2_ detection, Sur et al. [28] reduced the influence of fluctuations in interfering species on harmonics by selecting the optimal modulation depth, resulting in the requirement for the WMS background of water generated under typical combustion conditions. Compared with this approach, the present strategy reduces the sensitivity of harmonics to H_2_O based on the LCF-WMS-4*f* method and quantifies the effect of H_2_O by near-infrared H_2_O sensing.

## 3. Validation Experiment Setup

The combustion exhaust experiment setup consisted of the pitch side, catch side, auxiliary measurements, temperature-controlled long-path tube, burner facility, and flow control system. This section addresses the design of the multispecies TDLAS system.

### 3.1. Optical Setup

**Figure 6:**
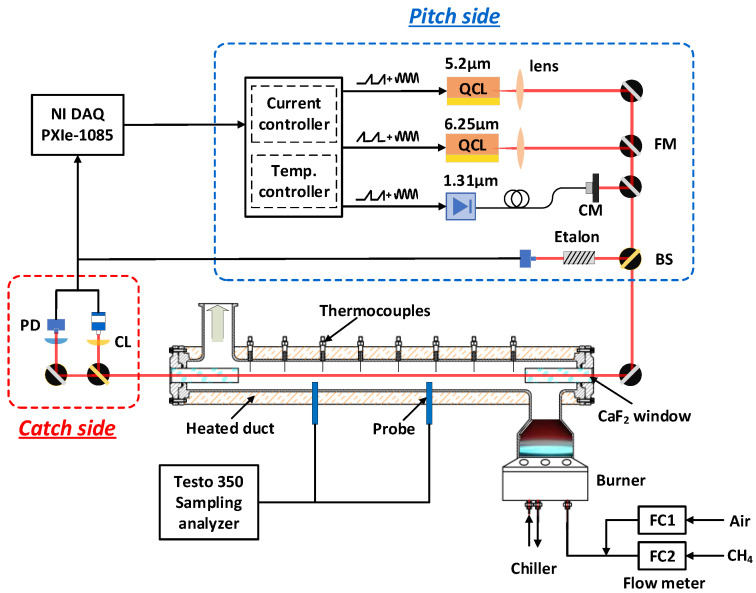
Schematic of the laser absorption diagnostics in an exhaust tube. FM, flipper mirror; BS—beam splitter; CM—collimator; PD—photodetector; CL—convex lens.

Figure 6 depicts the configuration of the test rig. For the pitch side, three continuous-wave lasers were chosen as the light source of the target gas diagnostics. Two mid-infrared QCLs (Alpes Lasers, Switzerland) mounted in the Laboratory Laser Housing package near 5.2 µm and 6.25 µm were utilized to detect the concentration of NO and NO_2_. Moreover, the light source for H_2_O detection was a narrow-linewidth, fiber-coupled near-infrared DFB laser (LD-PD, Sinpapore) near 1.31 µm. Laser controllers were used to provide stable and precise control of the laser diodes. The laser current was tuned by a 50 Hz scanning sawtooth signal superimposed on a 50 kHz sinusoidal modulation signal generated by the National Instruments DAQ system. The three laser beams were combined using flipped mirrors.

For the catch side, the transmitted laser beam was split into two parts by a CaF_2_ beam splitter. Then, these two parts were separately focused on an HgCdTe thermo-electrically cooled MIR detector LabM-I-6 (Vigo, Poland) and an InGaAs amplified NIR photodetector PDA10CS2 (Thorlabs, United States). The absorption signals were recorded by the DAQ system at a sampling rate of 2 MHz. The relationship between the laser scanning time and frequency can be characterized by a silicon etalon placed in another laser path.

### 3.2. Exhaust Tube Hardware

The cylindrical exhaust tube provided a 157.5 cm effective optical length, with 25 cm CaF_2_ column windows at both ends of the combustion tube to reduce the influence of the non-uniform temperature. Moreover, thermal insulation of the exhaust tube was provided by wrapping it in heating cables and ceramic wool. During the experiment, a McKenna burner provided combustion emissions with a relatively stable composition. The gas flow rates of the fuel and air were controlled and monitored by two mass flow controllers (Bronkhorst, 1% accuracy). The air flow rate was maintained consistently, and the experimental conditions were changed by adjusting the fuel flow rate. The equivalence ratio of the premixed CH_4_/air flat flame varied from 0.73 to 0.99, as shown in Table 1. Additionally, a cylinder glass cover was placed around the plate flame to prevent influence from the surroundings.

The gas mixture in the tube was sampled by a gas analyzer (O_2_, CO, CO_2_, NO_x_, Testo 350), and the accuracy of the NO_x_ sensor was around ±5 ppm at low concentrations (less than 100 ppm). Several fine-wire K-type thermocouples (0.2% accuracy) with a diameter of 0.3 mm were installed in the temperature-controlled exhaust tube.

## 4. Results and Discussion

### 4.1. LCF-WMS Data Processing Procedure

**Figure 7:**
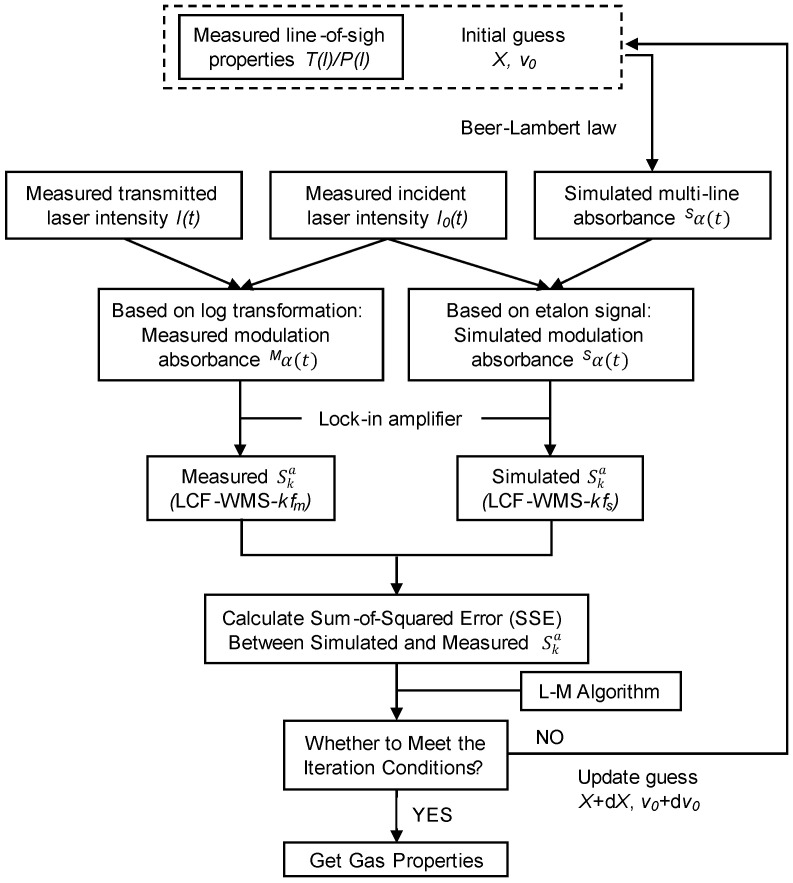
Algorithm for iterative fitting by comparing simulated and measured harmonic signals.

LCF-WMS-2*f* and LCF-WMS-4*f* signals were measured for H_2_O, NO, and NO_2_ transitions. Figure 7 shows the algorithm for the calculation of the concentration based on the LCF-WMS technique. The background laser intensity in high-purity nitrogen gas was recorded before each test to provide the background signal for the LCF-WMS measurements. The laser intensity recorded in the combustion gases and the background signal were passed through a digital lock-in filter combined with a low-pass filter and were applied to extract the harmonics for each laser. The normalized cutoff frequency of the low-pass filter was 0.00312, 0.00062, and 0.00031 for H_2_O, NO, and NO_2_, respectively. The actual gas properties can be inferred from the iterative fitting by comparing the simulation with the measured harmonic signals. It should be noted that the simulated multi-line absorbance considers the influence of both the target and interfering components with the estimation of the NO_x_ mole fraction and the known H_2_O concentration obtained through near-infrared H_2_O sensing.

### 4.2. Sensor Performance in Combustion Exhaust: A Demonstration of the Method

**Figure 8:**
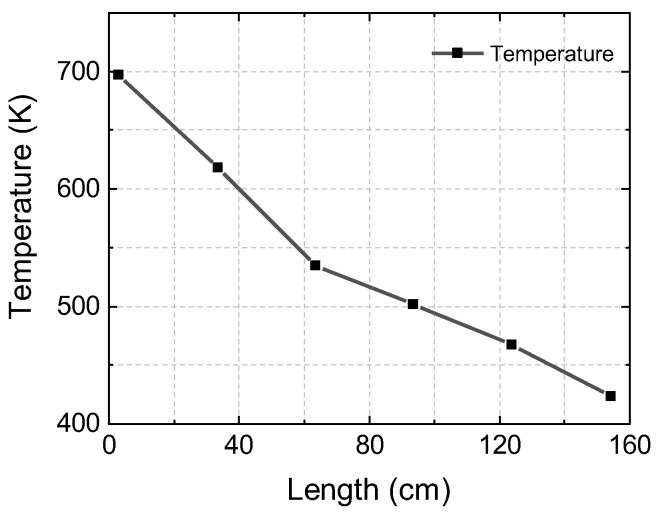
The temperature data along the optical length with typical experiment conditions at Φ = 0.89.

The heating temperature of the exhaust tube was set at 475 K. Temperatures were varied in the range of 430–700 K, which was measured by several thermocouples axially placed along the exhaust tube. Temperature data obtained at a steady state of Φ = 0.89 are plotted in Figure 8, indicating that the temperature gradient was present along the length of the exhaust tube due to the limitation of the thermal insulation materials. As the temperature varies along the LOS and the transition line strength is temperature-dependent, the path-integrated absorbance cannot be simplified by the uniform gas medium assumption. Existing knowledge of how gas conditions vary along the LOS can be a resolution for non-uniform measurement. In this work, the temperature profile was obtained by the polynomial fitting of the thermocouple data.

**Figure 9:**
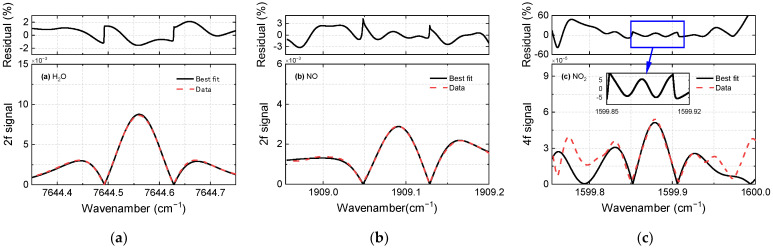
Measured (red dashed line) and best-fit (black solid line) harmonic line shapes for (**a**) H_2_O, (**b**) NO, and (**c**) NO_2_ in a combustion exhaust at Φ = 0.89.

Figure 9 shows the 2*f* line shape of H_2_O (a) and NO (b) in a representative combustion exhaust (Φ = 0.89). There is excellent agreement in the line shapes of the simulated and measured 2*f* signals. The relative fitting residuals (ratio of the peak value to the residual) were about 2% and 3% for H_2_O and NO, respectively. Figure 9c shows the 4*f* line shape of NO_2_ measured in the combustion exhaust and its corresponding best-fit curves. In the line shape of the central lobe, there is better agreement between the simulation and measurement than in the other parietal lobes. The concentration of NO_2_ was quantified using the central lobe, which represents the spectral region between 1599.85 cm^−1^ and 1599.92 cm^−1^. A higher discrepancy in the outer lobes can be observed, which is caused by vulnerability to water interference [28]. Accurate NO_2_ sensing requires an accurate characterization of the spectral parameters of the local water features. However, it is particularly difficult because the water transitions are weak and have high internal energy. Further work is needed for the accurate measurement of these water transitions.

The evaluation of uncertainty is important to reflect the reliability of the measurement data. The uncertainty in TDLAS measurements includes contributions from optical parameters and experimental noise [45,46]. In this research, the uncertainties in the optical parameters and temperature measurements along the LOS were transferred to the measurement results through the fitting process. The noise of the detector, as well as the laser fluctuation due to the instability of the laser temperature and laser current controller, results in systematic biasing and random noise.

**Figure 10:**
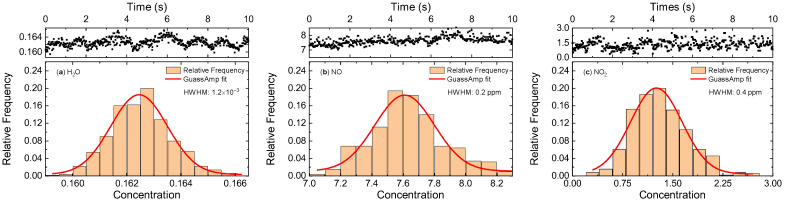
Frequency distribution of the measured data along with the Gaussian fitting based on the time-resolved measurements of (**a**) H_2_O, (**b**) NO, and (**c**) NO_2_ at Φ = 0.89.

Taking the NO_2_ measurement at Φ = 0.89 as an example, the fitting residual of the selected lines was less than 5% (1599.9 cm^−1^). To evaluate the random noise level of optical measurements, continuous recordings were performed at a relatively stable combustion state for 10 s. The frequency distribution of the measured data and the Gaussian profile-fitting curves are shown in Figure 10. The half width at half maximum (HWHM) of H_2_O, NO, and NO_2_ was 0.0012, 0.23 ppm, and 0.45 ppm, corresponding to a relative instrument accuracy of 0.76%, 2.93%, and 34.75%, respectively. The line-strength uncertainty of the absorption lines from the HITRAN database was ~10% [47]. The accuracy of the thermocouples was ~0.2%. These parameters were uncorrelated sources of uncertainties; thus, a total uncertainty of ~36% was obtained, leading to a concentration uncertainty of 0.47 ppm.

The sensor measurement resolution can be reflected by the 2 s noise, which was calculated using the values obtained from the RMS noise. Despite the H_2_O interference having been eliminated as much as possible, the 2 s noises of the NO_2_ measurement were estimated to be ~1 ppm. The NO_x_ generation decreased with the further reduction in the equivalence ratio due to the lower adiabatic flame temperature. Therefore, it was not efficient to measure NO_2_ at Φ = 0.78 and 0.73 since the random noise was higher than the predicted concentration. Extra data about the concentrations and uncertainties of these three components are listed in Table 2. The flame number and the corresponding equivalence ratio of the flat flame are also listed.

### 4.3. Comparison and Validation of Experimental Results

**Figure 11:**
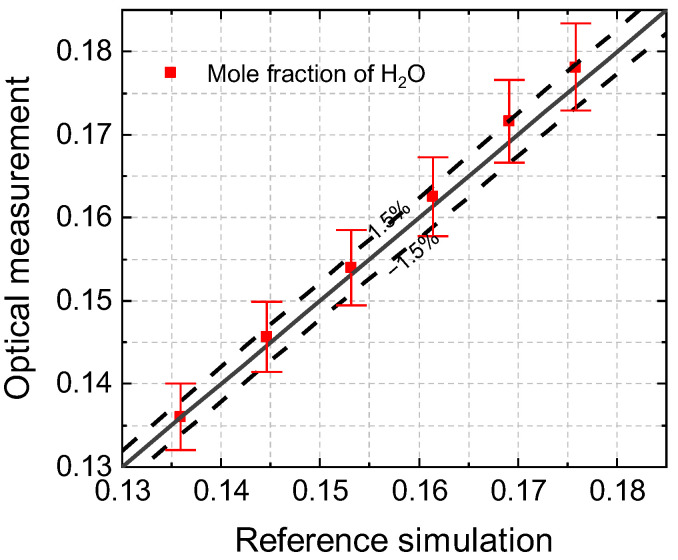
Mole fraction of H_2_O in combustion exhaust gas measured using absorption spectroscopy versus equilibrium calculation using Chemkin with an equivalence ratio of 0.99–0.73.

Under the assumption of complete combustion, temperature changes caused by the heat loss effect have negligible impacts on the mole fraction of H_2_O generated from the combustion process. The measurements of H_2_O and the simulation results calculated using Chemkin are described in Figure 11. Additionally, the deviation of both data was less than 1.5% in all experimental conditions. This proves that the optical method was effective in achieving a quantitative measurement in non-uniform flow fields.

As described in Section 4.2, ultra-low NO_x_ emissions were captured by both the optical measurement and the reference sampling measurement. According to previous research, the generation of NO_x_ can be affected by the heat loss ratio. The definition of the heat loss ratio and both experimental and simulation results have been published [48]. In the presence of the radiation from the combustion zone to the surrounding environment and the forced heat dissipation by the chiller, the effect of heat loss from the burner outlet to the exhaust tube inlet cannot be ignored in this research, since it led to the NO_x_ emission being ultra-low. Despite the fact that the total heat loss ratio could be obtained using the previous method, the proportion of radiation and the forced heat dissipation were less clear. Moreover, the maximum temperature in the combustion zone was influenced by the forced heat dissipation. Therefore, accurate NO_x_ values were difficult to obtain from the calculation.

The TDLAS results of NO and NO_2_ were compared with the gas analyzer results. The deviation between the two methods may be due to the following reasons: (1) There is an assumption in the data process that the distribution of concentration throughout the range is uniform. However, NO_2_ begins dissociating near 423 K. Therefore, there may be a concentration gradient of NO_x_ dissociation along the LOS. (2) The measured position of the sampling method is not exactly the same as the non-invasive optical method. (3) It must be clarified that the measurement uncertainty of the Testo 350 is higher than the predicted sensor accuracy in the CLD analyzer (±5 ppm) due to the loss of the condensate and pipeline absorption.

**Figure 12:**
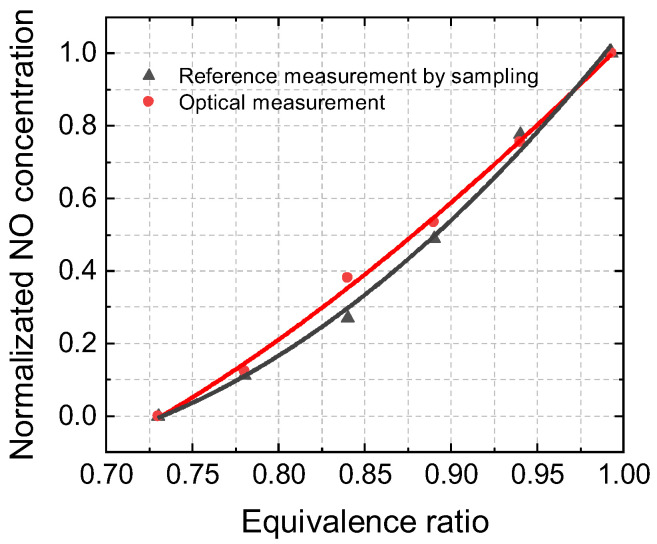
Normalized NO concentration in combustion gas measured by optical measurement and a gas analyzer in a premixed flat flame with an equivalence ratio of 0.99–0.73.

**Figure 13:**
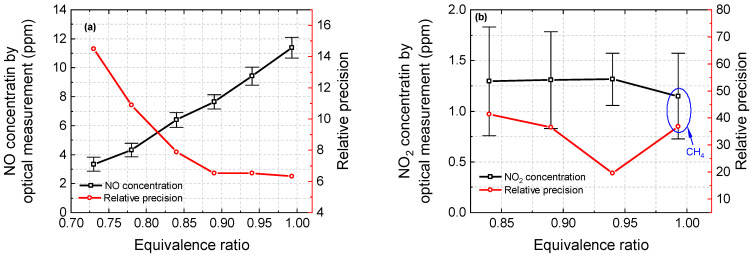
Measured NO (**a**) and NO_2_ (**b**) concentrations and relative precision varying with the equivalence ratio in combustion gases.

The relative change in the NO concentration under different experimental conditions can be reflected in the data recorded by the gas analyzer. The normalized NO emission trends between the optical measurement and Testo 350 were similar, as shown in Figure 12. As shown in Figure 13, NO (a) and NO_2_ (b) concentrations as well as measurement precisions decreased with the equivalence ratio, except for the NO_2_ measurement under the operation condition of Φ = 0.99. It is worth noting that when the experimental conditions are close to the chemical equivalence ratio, the residue of methane could affect the measured NO_2_ concentration and lead to a reduction in measurement accuracy. Therefore, experiments are recommended to be performed under fuel-lean conditions. Further verification requires the measurement of the unburned hydrocarbon content (UHC).

## 5. Conclusions

In this work, multispecies quantitative measurements of trace NO and NO_2_ with strong broad spectral H_2_O interference were demonstrated in combustion exhaust at high temperatures. The proposed strategy was based on the scanned-wavelength LCF-WMS method combined with temperature profile fitting. A discussion of the transition line selection procedure and the trace gas detection methodology in the presence of a significant amount of gaseous water was presented. Absorption transitions within the near-infrared (~1308 nm) and mid-infrared (~6250 nm and ~5238 nm) spectra were probed for accurate and sensitive concentration measurements.

Experiments were conducted at different methane mass fluxes, corresponding to an equivalence ratio Φ ranging from 0.73 to 0.99. The temperature variation along the optical path was provided by thermocouples. In the presence of a significant temperature gradient, the measured H_2_O mole fractions were found to be in agreement with the numerical simulation data calculated using Chemkin (less than 1.5%). Both optical measurements and traditional sampling data showed ultra-low NO_x_ emissions. Under different combustion conditions, 2s noise levels were observed to deteriorate from 0.4 to 1.0 ppm, 0.5 to 0.9 ppm, and 1.9 to 2.4 ppm, corresponding to a relative precision level of 2.4–13.3%, 16.1–39.8%, and 0.6–0.8%, for NO, NO_2_, and H_2_O, respectively. The measurement precision and NO_x_ concentrations decreased with the equivalence ratio. The emerging water vapor lines with high internal energy and ultra-low NO_x_ concentrations were the main constraints in the measurement accuracy. In addition, CH_4_ affected the accuracy of the NO_2_ measurement. Experiments are recommended to be performed under fuel-lean conditions.

According to the case studied in combustion emissions, the developed measurement technique was demonstrated to be reliable and appropriate for providing accurate detection of low-absorbance NO and NO_2_ with high H_2_O interference. This method can be improved in terms of the spatial and temporal resolution and has the potential to be used for monitoring the combustion exhaust from gas turbines.

## Figures and Tables

**Table 1:** Experimental conditions of the premixed CH_4_/air flame in this work.

Flame No.	CH_4_ (L/min)	Air (L/min)	Φ
1	1.65	16.5	0.99
2	1.56	16.5	0.94
3	1.47	16.5	0.89
4	1.39	16.5	0.84
5	1.30	16.5	0.78
6	1.21	16.5	0.73

**Table 2:** Species mole fractions measured by TDLAS are listed beside the reference values calculated using Chemkin or measured by a gas analyzer.

Flame No.	*X*_H2O_ Chemkin/Laser Abs	*X*_NO_ (ppm) Testo 350/Laser Abs	*X*_NO2_ (ppm) Testo 350/Laser Abs	Φ
1	0.1759/0.1781 ± 0.0052	19.8 ± 5/11.39 ± 0.72	3.0 ± 5/1.15 ± 0.42	0.99
2	0.1692/0.1716 ± 0.0050	16.4 ± 5/9.43 ± 0.61	3.5 ± 5/1.32 ± 0.26	0.94
3	0.1614/0.1625 ± 0.0047	11.9 ± 5/7.64 ± 0.49	2.9 ± 5/1.30 ± 0.47	0.89
4	0.1531/0.1539 ± 0.0045	8.6 ± 5/6.40 ± 0.50	2.6 ± 5/1.29 ± 0.54	0.84
5	0.1447/0.1457 ± 0.0042	6.2 ± 5/4.32 ± 0.47	2.3 ± 5/-	0.78
6	0.1359/0.1361 ± 0.0039	4.5 ± 5/3.33 ± 0.48	1.9 ± 5/-	0.73

## Data Availability

Not applicable.
